# How Have We Diagnosed Early-Stage Lung Cancer without Radiographic Screening? A Contemporary Single-Center Experience

**DOI:** 10.1371/journal.pone.0052313

**Published:** 2012-12-21

**Authors:** Evelyn O. Taiwo, Jeffrey T. Yorio, Jingsheng Yan, David E. Gerber

**Affiliations:** 1 Department of Internal Medicine, University of Texas Southwestern Medical Center, Dallas, Texas, United States of America; 2 Division of Hematology-Oncology, University of Texas Southwestern Medical Center, Dallas, Texas, United States of America; 3 Department of Clinical Sciences (Division of Biostatistics), University of Texas Southwestern Medical Center, Dallas, Texas, United States of America; 4 Harold C. Simmons Cancer Center, University of Texas Southwestern Medical Center, Dallas, Texas, United States of America; University of Nebraska Medical Center, United States of America

## Abstract

**Background:**

The National Lung Screening Trial (NLST), which demonstrated a reduction in lung cancer mortality, may result in widespread computed tomography (CT)-based screening of select populations. How early-stage lung cancer has been diagnosed without screening, and what proportion of these cases would be captured by a screening program modeled on the NLST, is not currently known. We therefore evaluated current patterns of early-stage lung cancer presentation.

**Methodology/Principal Findings:**

We performed a single-institution retrospective analysis of patients diagnosed with stage I–II non-small cell lung cancer (NSCLC) from 2000–2009. Associations between patient and imaging characteristics were assessed using univariate and multivariate analyses. A total of 412 patients met criteria for analysis. Among those with available reason for initial imaging, the reason was symptoms in 51%, follow-up of other conditions in 43%, and screening in 6%. Reason for imaging was associated with race (*P*<0.001), insurance type (*P* = 0.005), and disease stage (P<0.001). Type of initial imaging was associated with reason for imaging (*P*<0.001), year (chest x-ray 67% in 2000–2004 vs. 49% in 2005–2009; *P*<0.001), and disease stage (*P* = 0.005). Among patients with available quantified smoking history, 48% were age 55–74 years and smoked 30-plus pack-years, therefore meeting NLST entry criteria.

**Conclusions/Significance:**

Symptoms remain a dominant but declining reason for detection of early-stage NSCLC. The proportion of cases detected initially by CT scan without antecedent chest x-ray has increased considerably. Because as few as half of cases meet NLST eligibility criteria, clinicians should remain aware of the diverse circumstances of early-stage lung cancer presentation to expedite therapy.

## Introduction

Disease presentation at advanced stage remains a key factor in the poor outcomes of lung cancer. Underlying the challenges of detecting and diagnosing lung cancer earlier in the disease course are lack of specific associated symptoms and lack of established screening approaches. Typical clinical features of lung tumors—such as cough, dyspnea, and chest pain—may not develop until disease is more locally advanced, or may be attributed to more common, non-malignant etiologies. While efforts to screen high-risk, asymptomatic individuals for lung cancer date back decades, [1], [2] it is only with the recently published National Lung Screening Trial (NLST) that a reduction in lung cancer mortality has been shown. [3], [4] This randomized controlled trial, which enrolled over 50,000 subjects ages 55–74 years who smoked 30 or more pack-years, compared three annual low-dose helical computed tomography (CT) scans with three annual chest x-rays (CXR). At the time of study closure (median follow-up 6.5 years, maximum follow-up 7.4 years), those screened with low-dose spiral CT had a 20% reduction in lung cancer deaths and a 7% reduction in all-cause mortality compared to subjects in the CXR arm.

With the recent endorsement of professional organizations such as the American Lung Association, the American Cancer Society (ACS), and the National Comprehensive Cancer Network (NCCN), [5], [6] widespread radiographic screening for lung cancer is likely to be implemented. This possibility raises two key questions: What are the current reasons for and methods of diagnosis of early-stage lung cancer? What proportion of these cases will be captured by a screening program modeled on the NLST? Even without reliable, specific symptoms or established screening programs, it is estimated that 17–25% of lung cancer cases are diagnosed at stage I. [7], [8] How these cases currently come to medical attention and are diagnosed is not known, as most studies describing the presentation of lung cancer were performed 25–45 years ago, a time when almost all cases were detected by CXR ordered to evaluate cardiopulmonary symptoms. [9]–[14] We therefore evaluated the presentation of early-stage lung cancer in a contemporary population, with particular focus on the type and reason for initial imaging studies. Of these cases, we determined which would have been eligible for the NLST.

## Methods

### Ethics Statement

This study was approved by the University of Texas Southwestern Medical Center (UT Southwestern) Institutional Review Board. The Institutional Review Board waived the requirement for informed consent for the following reasons: (1) the research involved no more than minimal risk to subjects (in this case compilation of data and subsequent risk of loss of confidentiality); (2) the waiver did not adversely affect the rights and welfare of the subjects (in this case no treatment or invasive procedures were involved, and collected data were disclosed only for analytical purposes); (3) the research could not practicably be carried out without the waiver (in this case, a retrospective medical records review of a large volume of cases from a time period starting more than 10 years prior to the analysis).

### Study Setting

The study sample was obtained from the UT Southwestern-associated clinical facilities, which include Parkland Health and Hospital System and University Hospital. Parkland comprises a 968-bed safety net hospital and associated community clinics that provide care to the indigent and uninsured population of Dallas County. University Hospital is a 415-bed inpatient facility and outpatient clinics providing primary and specialty medical and surgical care. During the study period, these institutions did not participate in radiographic lung cancer screening studies. These clinical sites are located in Dallas, Texas. Dallas County is the eighth most populous county in the United States, with an estimated 2.4 million residents, of whom 39% are Hispanic, 35% are non-Hispanic white, and 21% are African American. [15]

### Data Extraction and Study Population

We collected data on consecutive patients diagnosed with stage I and II NSCLC between January 1, 2000, and December 31, 2009, from the UT Southwestern and Parkland tumor registries, which abstract data directly from medical records according to standards established by the American College of Surgeons Commission on Cancer, Surveillance Epidemiology and End Results (SEER)/National Cancer Institute (NCI), and the National Program of Cancer Registries. We obtained additional information as needed from individual patient electronic medical records. For each case, the following data were recorded: patient age, gender, race/ethnicity, health insurance type, smoking status and duration; type of initial imaging, reason for imaging (as recorded in the imaging study requisition); tumor stage, histology, and location.

We limited the study population to stage I and II NSCLC because these represent the predominant stages detected in earlier screening trials. [16] The 2000–2009 study period was selected because adequate data were first recorded by the tumor registries in 2000 and, at the time of our analysis, registry data collection was complete through 2009.

### Statistical Analysis

All statistical analyses were performed using SAS 9.2 (SAS Institute, Cary, North Carolina) in Microsoft Windows. Descriptive statistics were generated for baseline case characteristics. Univariate and multivariable logistic regression models were used to explore the association between case characteristics, reason for imaging, and type of imaging. In the multivariate analyses, models were selected based on significance of association in univariate analysis and data availability. Analysis of Kaplan-Meier curves was used to compare overall survival according to reason for and type of imaging.

## Results

### Study Population

A total of 412 patients met study criteria. Data on age, gender, race, and tumor histology were available for all patients. Mean age was 67 years, 49% were men, 70% were white, and 79% had stage I disease. Additional case characteristics are listed in **Table 1**. Smoking status (characterized as current/former/never) was available for 333 patients (81%), and full pack-year data was available for 267 patients (65%). Baseline characteristics of cases with and without complete smoking history were similar: median age was 69 years vs. 67 years; racial composition was 67% and 70% white, respectively.

**Table 1:** Baseline Case Characteristics.

Characteristics	Mean ± SD or Number (%)
No. of patients	412
Age at diagnosis (years)	66.8±10.5
**Gender**	
Male	201 (49)
Female	211 (51)
**Race**	
White	288 (70)
Black	94 (23)
Hispanic	12 (3)
Other	18 (4)
**Insurance**	
Private	111 (27)
Medicare	230 (56)
Indigent^a^	58 (14)
Unknown	13 (3)
**Smoking status**	
Current	162 (39)
Former	153 (37)
Never	18 (5)
Unknown	79 (19)
**Smoking duration** b	
<30 pack-years	63 (24)
≥30pack-years	204 (76)
**Reason for imaging**	
Symptoms	158 (38)
Screening	20 (5)
Other indication	132 (32)
Unknown	102 (25)
**Symptom Type** c	
Cough/hemoptysis	54 (36)
Dyspnea	46 (29)
Chest pain	24 (15)
Other	34 (20)
**Image type**	
CXR	223 (54)
CT	156 (38)
Other	10 (2)
Unknown	23 (6)
**Year of diagnosis**	
2000–2004	196 (48)
2005–2009	216 (52)
**Histology**	
Squamous	123 (30)
Adenocarcinoma	213 (52)
Other	76 (18)
**Clinical Stage**	
Stage I	323 (78)
Stage II	89 (22)

a Includes Medicaid, county health plan, and no insurance.

b Of 267 cases with full smoking history data.

c Of 158 cases presenting with symptoms.

CXR, chest x-ray; CT, computed tomography; SD, standard deviation.

### Reason for imaging

Of the 310 patients with an identified reason for imaging, 158 patients (51%) had imaging performed to evaluate symptoms. Almost all symptoms were cardiopulmonary in nature: cough/hemoptysis (36%), dyspnea (29%), chest pain (15%). Among cases categorized as “cough/hemoptysis” (N = 54), 21 (7% of all cases) presented with hemoptysis. Abdominal pain was the symptom under evaluation in 5% of cases. Imaging was performed for follow-up of other, non-malignant disease in 70 patients (23%), follow-up of another cancer in 52 patients (17%), lung cancer screening in 20 patients (6%), and pre-operative evaluation in 10 patients (3%). The 52 cases with imaging performed to evaluate a prior malignancy included the following specific cancer types: head and neck (18), lung (12), breast (9), bladder (3), prostate (2), lymphoma (2), cervical (1), skin (1), ovarian (1), pancreas (1), kidney (1), thyroid (1).

Of the 20 cases with imaging performed for cancer screening, median age was 71 years, 11 (55%) were women, and smoking history was as follows: 6 current smokers, 11 former smokers, and 3 unknown. The average duration of smoking was 48 pack-years. Initial imaging was CXR for 16 of these 20 cases, with the remainder CT scans.

**Figure 1:**
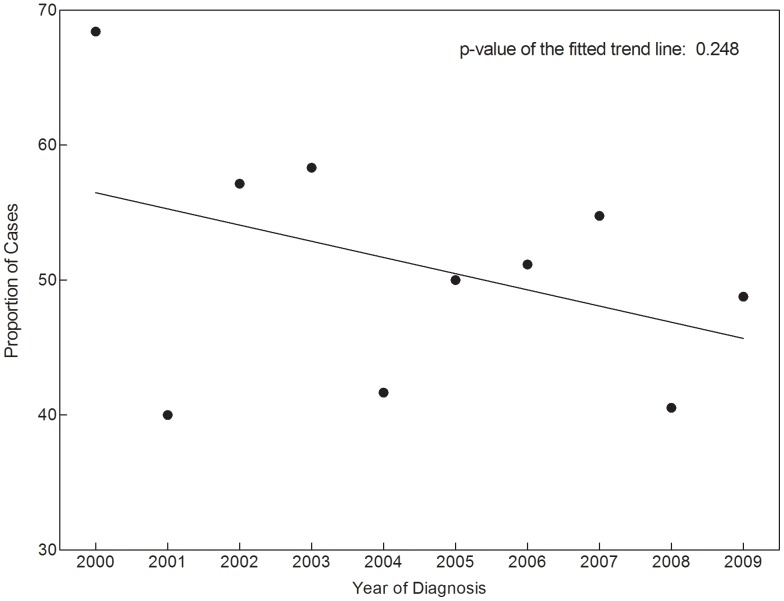
Proportion of early-stage NSCLC cases undergoing initial imaging to evaluate symptoms.

In univariate analysis, reason for initial chest imaging (dichotomized as symptom evaluation versus other) was significantly associated with race, insurance type, image type, and clinical stage (see **Table 2**). Over time, there was a decrease in symptom evaluation as the reason for imaging (see **Figure 1**), but this trend was not statistically significant (*P* = 0.25). In a multivariable model that included race, insurance type, image type, and clinical stage, reason for imaging had a trend toward association with race, with non-white patients more likely to be imaged to evaluate symptoms (HR 1.71; 95% CI, 0.94–3.10; *P* = 0.08). Image type and clinical stage were significantly associated with reason for imaging. Cases initially identified by non-CXR techniques were less likely to have undergone imaging to evaluate symptoms (HR 0.36; 95% CI, 0.21–0.60; *P*<0.001). Stage II cases were more likely than stage I cases to be imaged initially to evaluate symptoms (HR 4.43; 95% CI, 2.16–9.10; *P*<0.001).

**Table 2:** Association between case characteristics and reason for imaging (univariate analysis).

	Patients undergoing imaging to evaluate symptoms (%)	OR (95% CI) for imaging performed to evaluate symptoms	Overall *P* value
**Age**			
≤65 years	54	1.25 (0.79–1.95)	0.34
>65 years	49	Reference	
**Gender**			
Male	51	0.98 (0.63–1.52)	0.91
Female	51	Reference	
**Race**			
White	44	Reference	<0.001
Non-white	65	2.43 (1.48–3.98)	
**Insurance**			
Private	55	Reference	
Medicare	44	0.64 (0.37–1.08)	0.005
Indigent^a^	71	1.95 (0.89–4.27)	
**Smoking status**			
Current	54	Reference	0.06
Former/never	43	0.64 (0.39–1.03)	
**Imaging type**			
CXR	64	Reference	<0.001
Other	34	0.28 (0.18–0.46)	
**Year of diagnosis**			
2000–2004	55	1.25 (0.79–1.98)	0.35
2005–2009	49	Reference	
**Histology**			
Adenocarcinoma	52	Reference	
Squamous cell	51	0.96 (0.58–1.61)	0.89
Other	48	0.87 (0.42–1.57)	
**Clinical stage**			
1	43	Reference	<0.001
2	79	5.05 (2.66–9.59)	

**OR >1: More likely to have imaging performed for evaluation of symptoms than for other reasons.**

a Includes Medicaid, county health plan, and no insurance.

CI, confidence interval; CXR, chest x-ray; OR, odds ratio.

### Type of imaging

**Figure 2:**
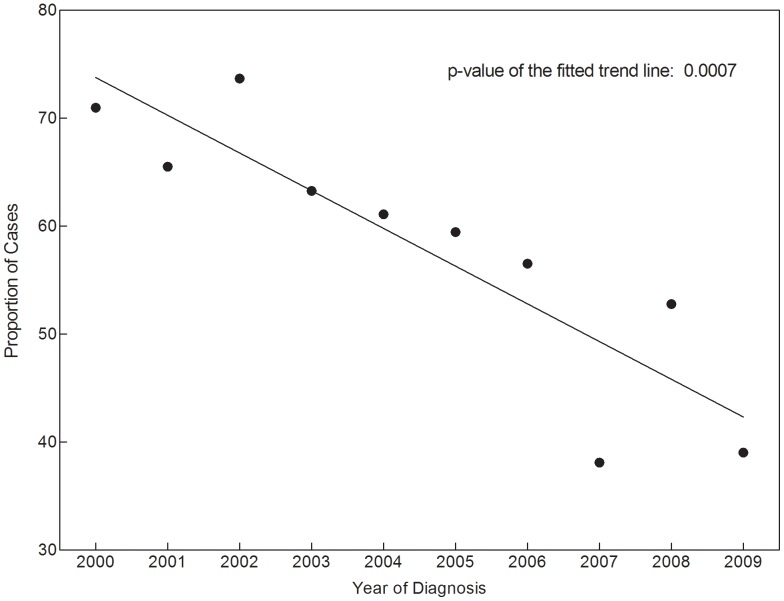
Proportion of early-stage NSCLC cases initially detected by chest x-ray.

Among the 389 cases with identified initial imaging studies, 57% were CXR, 40% were chest CT, and 3% were other. The “other” cases included 9 positron emission tomography (PET) scans and 1 cardiac magnetic resonance imaging scan. In univariate analysis, image type was significantly associated with smoking status, reason for imaging, year of diagnosis, and clinical stage (see **Table 3** and **Figure 2**). In a multivariable model including gender, race, insurance, reason for imaging, year of diagnosis, and clinical stage, initial image type remained associated with reason for imaging and year of diagnosis. Cases detected for reasons other than symptoms were less likely to have an initial CXR (HR 0.36; 95% CI, 0.21–0.62). Cases diagnosed 2000–2004 were more likely to have had an initial CXR than were cases diagnosed 2005–2009 (HR 2.63; 95% CI, 1.54–4.51; *P*<0.001).

**Table 3:** Association between case characteristics and type of initial imaging (univariate analysis).

	Patients undergoing CXR (%)	OR (95% CI) for CXR	Overall *P* value
**Age**			
≤65 years	58	1.05 (0.70–1.58)	0.81
>65 years	57	Reference	
**Gender**			
Male	61	1.39 (0.93–2.09)	0.11
Female	53	Reference	
**Race**			
White	55	Reference	0.11
Non-white	63	1.43 (0.92–2.23)	
**Insurance**			
Private	62	Reference	
Medicare	53	0.69 (0.43–1.11)	0.13
Indigent^a^	66	1.18 (0.59–2.35)	
**Smoking status**			
Current	63	Reference	0.03
Former/never	51	0.61 (0.39–0.96)	
**Imaging reason**			
Symptoms	70	Reference	<0.001
Other	40	0.28 (0.18–0.46)	
**Year of diagnosis**			
2000–2004	67	2.14 (1.41–3.23)	<0.001
2005–2009	49	Reference	
**Histology**			
Adenocarcinoma	57	Reference	
Squamous cell	61	1.17 (0.74–1.86)	0.39
Other	51	0.77 (0.45–1.33)	
**Clinical stage**			
1	54	Reference	0.005
2	71	2.16 (1.26–3.69)	

**OR >1: more likely to have CXR than other modalities as initial imaging study.**

a Includes Medicaid, county health plan, and no insurance.

CI, confidence interval; CXR, chest x-ray; OR, odds ratio.

### Comparison with NLST population

Among all 412 patients in the study cohort, 154 patients (37%) would have been ineligible for the NLST because of age: 55 patients were under age 55 years; 99 patients were over age 74 years. Sixty-three of the 412 patients (15%) would be ineligible due to inadequate smoking history (<30 pack-years). Among the 258 patients (63%) age 55–74 years, 35 smoked less than 30 pack-years. Thus, among all 412 patients in the cohort, 189 (46%) would have would have been ineligible for the NLST.

Limiting the analysis to the 267 patients in the cohort for whom quantified smoking history was available, 103 patients (39%) would be ineligible for the NLST because of age: 42 patients were under age 55 years; 61 patients were over age 74 years. Sixty-three of these 267 patients (24%) would have been ineligible for the NLST due to total smoking history less than 30 pack-years. Among the 164 patients age 55–74 years, 35 smoked less than 30 pack-years. Thus, among the 267 patients with available quantified smoking history, 138 (52%) would have been ineligible for the NLST. Twenty-eight cases (11%) failed to meet either smoking or age criteria, of which 15 were under age 55 years and 13 were over age 74 years. Removing cases diagnosed because of CXR screening or because of radiographic follow-up of a prior cancer diagnosis from the analyses did not meaningfully change these results (data not shown).

## Discussion

Detection of lung cancer at early and more treatable stages has been a principal focus of researchers and clinicians for decades, culminating in the recently announced positive results of the NLST. In spite of these efforts, little is known about how—in the absence of widespread screening programs—patients with stage I–II disease currently present and are diagnosed. To our knowledge, this is the first study to examine the reasons for and methods of early-stage lung cancer diagnosis in a contemporary setting.

Symptom evaluation was the most common reason for initial imaging study. However, accounting for approximately half of cases in this series, this rate is far lower than previously reported. In a 1980 Finnish population-based study, almost all cases were detected due to symptoms, with only 12% noted incidentally during examination for other disease. [9] An earlier study from Finland found that only 6% of early-stage lung cancer cases did not have associated symptoms at presentation. [14] For individual cases in this study, it is not known whether symptoms were related to the subsequently diagnosed malignancy or occurred incidentally. However, the observation that stage II cases were more likely than stage I cases to undergo initial imaging because of symptoms (79% versus 43%) suggests that a proportion of symptoms do indeed arise from the lung tumor. In univariate analysis, symptom-related presentation was more common among non-white and indigent patients. A possible explanation is, due to less access to routine healthcare, these patients had a lower likelihood of undergoing serial imaging as follow-up for other medical conditions.

Of note, in the absence of evidenced-based recommendations at the time, 5% of patients in this series had initial imaging performed for the purpose of lung cancer screening. These cases were heavy smokers and almost all were imaged initially by CXR. From our data, it is not possible to determine the prevalence of this practice. We considered the possibility that cases with missing reason for imaging (25% of cases) were also performed for the purpose of screening, with the ordering physician intentionally omitting a study indication on the requisition form. Based on patterns of availability of other data, this seems unlikely. The identity of the ordering physician was recorded in 198 of 412 cases (48%) in our overall cohort. However, the identity of the ordering physician was recorded in only 2 of 102 cases (2%) in which the reason for imaging was not recorded. Furthermore, these 102 cases come disproportionately from early years in our study period (almost all 2000–2003). Accordingly, we believe cases with missing reasons for imaging reflect the nature of clinical data recording in our institutional electronic medical record at the time the imaging studies were performed, rather than clinicians' actively choosing not to specify this information.

Over the 10 recent years covered in this study, the proportion of lung cancer cases initially detected by CT scan without antecedent CXR increased over 50%. This trend is consistent with other reports of rising rates of CT use. Larson and colleagues reported a 16% annual increase in CT ordering in an emergency department. [17] CT-detected cases were also more common when imaging was ordered for non-symptom-related reasons, the patient was a former or never smoker (rather than a current smoker), and the case was eventually classified as stage I. It is not known whether recent reports describing the risks of diagnostic radiation exposure will counter this rising use of CT scans. [18], [19]

Comparing our population to that eligible for clinical trials of radiographic screening provides insight into how implementation of screening guidelines might impact current patterns of early-stage disease presentation. To optimize the use and benefit of screening, screening studies have limited enrollment to variably defined high-risk populations. The Early Lung Cancer Action Project (ELCAP) trial included individuals age ≥60 years with ≥10 pack-years smoking, while the Mayo Lung Project (MLP) trial enrolled men age ≥45 years who smoked at least 1 pack per day. [1], [2], [20] In the NLST, eligible participants were ages 55–74 years, had a smoking history of at least 30 pack-years, and, if former smokers, had quit within the previous 15 years. Individuals with a history of another cancer (apart from non-melanoma skin cancer and carcinoma *in situ*) within the past 5 years or any history of lung cancer were excluded. [3]

Applying NLST eligibility criteria to our population, approximately half of patients would have qualified for screening. This is likely an overestimate, as our analysis does not account for patients' prior cancer history (at least 17% of our cohort had a prior malignancy) or the timing of quitting for former smokers. Our results suggest that a substantial proportion of patients currently presenting with early-stage NSCLC would continue to do so independently of radiographic screening if such a program were implemented according to NLST criteria. Indeed, the possibility of frequent detection of early-stage disease outside of a screening context seems more likely with lung cancer than with other malignancies, as chest imaging is a more common practice in non-screening clinical care than are mammograms, Pap smears, and colonoscopies.

Importantly, it has been suggested that many clinicians might be inclined to apply radiographic lung cancer screening to a broader population in actual clinical practice. [21] In our series, age was the most common reason for cases failing to meet NLST criteria, with almost two-thirds of these patients older than the age cut-off of 74 years. This pattern raises questions about stopping cancer screening once a specific age threshold is reached, a complex issue for patients and physicians alike. [22], [23] In a recent study, octogenarians with early-stage lung cancer deemed appropriate for surgery tolerated lobectomy well. [24] These nuanced considerations are reflected in recent versions of the National Comprehensive Cancer Network (NCCN) guidelines, which do not specify a maximum age for lung cancer screening among high-risk individuals. [25] Similar observations have been made in the Rotterdam Study. [26], [27] In that contemporary prospective cohort of individuals age 55 years and older, only 30% of incident lung cancer cases would have met criteria for the NLST. Almost 60% were age greater than 74 years, and 13% were age 55–74 but smoked less than 30 pack-years.

Our findings raise the possibility that radiographic screening might be investigated in other well-defined populations, such as individuals with a prior history of malignancy (17% of our cohort). For instance, while radiographic surveillance is generally recommended for up to 5 years after definitive treatment of lung cancer, according to NLST eligibility criteria, this population would not qualify for annual chest CT scans thereafter. However, these patients face an ongoing heightened risk of future cancers. In our series, of the 52 cases diagnosed because of imaging performed to follow-up a prior cancer diagnosis, 35 (67%) had a potentially tobacco-associated malignancy (head and neck, lung, bladder, cervical, pancreatic). Additionally, it is possible that some of the 11 cases (21%) of breast cancer and lymphoma may have received prior chest irradiation, placing them at increased risk for lung cancer. Patients presenting with symptoms also require careful consideration. By definition, radiographic evaluation of these individuals does not constitute screening. Nevertheless, given the prevalence of symptoms at the time of presentation in our cohort (51%), it seems plausible that early radiographic evaluation of cardiopulmonary complaints in a high-risk population might result in earlier detection of lung cancer. What that evaluation should entail, and what should trigger it, remains unclear.

Principal limitations of this study include a relatively small sample size, a single-center setting, and the degree of missing data for certain variables, particularly smoking history and reason for imaging. Indeed, it is interest in these variables, which are not typically recorded by tumor registries, that precludes performing such a study in a large administrative dataset. Furthermore, a number of studies have suggested that, even when reported, the consistency and reliability of smoking data are variable. [28], [29] Our study setting, an academic medical center in the southern United States, may limit generalizability, although our cohort is racially and socioeconomically diverse. The exclusion of small cell cases from this series is unlikely to be clinically meaningful, as this histologic type may account for less than 10% of cases detected by screening. [30]

In conclusion, this is the first study to examine patterns of presentation and diagnosis of early-stage lung cancer in a contemporary population without widespread radiographic screening. Symptom evaluation remains a dominant but declining reason for initial detection of early-stage NSCLC, followed by radiographic assessment of other diseases. Even in an era lacking supporting evidence, a small proportion of cases have been detected by radiographic studies—principally CXR—ordered to screen for lung cancer. The proportion of early-stage NSCLC initially detected by CT scan has increased considerably in recent years, reflecting the growing use of this imaging modality. If widespread lung cancer screening is implemented according to NLST criteria, these patterns may persist, as only approximately half of the cases in this series met NLST eligibility criteria. Determining whether screening efforts should be extended to these NLST ineligible populations would require demonstration of a reduction in lung cancer mortality in a prospective trial. Until then, clinicians should remain aware of the diverse reasons for and circumstances of early-stage lung cancer presentation to expedite further evaluation and potentially curative treatment.
